# Population Genomics Reveals Seahorses (*Hippocampus erectus*) of the Western Mid-Atlantic Coast to Be Residents Rather than Vagrants

**DOI:** 10.1371/journal.pone.0116219

**Published:** 2015-01-28

**Authors:** J. T. Boehm, John Waldman, John D. Robinson, Michael J. Hickerson

**Affiliations:** 1 Department of Biology, City College of New York, 160 Convent Ave., New York, New York, 10031, United States of America; 2 Biology Department, Queens College, City University of New York, 65-30 Kissena Blvd., Queens, New York, 11367-1597, United States of America; 3 Subprogram in Ecology, Evolution and Behavior, The Graduate Center of the City University of New York, 365 5^th^ Ave, New York, New York, 10016, United States of America; 4 South Carolina Department of Natural Resources, Marine Resources Research Institute, 217 Fort Johnson Rd., Charleston, South Carolina, 29412, United States of America; Leibniz-Institute of Freshwater Ecology and Inland Fisheries, GERMANY

## Abstract

Understanding population structure and areas of demographic persistence and transients is critical for effective species management. However, direct observational evidence to address the geographic scale and delineation of ephemeral or persistent populations for many marine fishes is limited. The Lined seahorse (*Hippocampus erectus*) can be commonly found in three western Atlantic zoogeographic provinces, though inhabitants of the temperate northern Virginia Province are often considered tropical vagrants that only arrive during warm seasons from the southern provinces and perish as temperatures decline. Although genetics can locate regions of historical population persistence and isolation, previous evidence of Virginia Province persistence is only provisional due to limited genetic sampling (i.e., mitochondrial DNA and five nuclear loci). To test alternative hypotheses of historical persistence versus the ephemerality of a northern Virginia Province population we used a RADseq generated dataset consisting of 11,708 single nucleotide polymorphisms (SNP) sampled from individuals collected from the eastern Gulf of Mexico to Long Island, NY. Concordant results from genomic analyses all infer three genetically divergent subpopulations, and strongly support Virginia Province inhabitants as a genetically diverged and a historically persistent ancestral gene pool. These results suggest that individuals that emerge in coastal areas during the warm season can be considered “local” and supports offshore migration during the colder months. This research demonstrates how a large number of genes sampled across a geographical range can capture the diversity of coalescent histories (across loci) while inferring population history. Moreover, these results clearly demonstrate the utility of population genomic data to infer peripheral subpopulation persistence in difficult-to-observe species.

## Introduction

**Figure 1:**
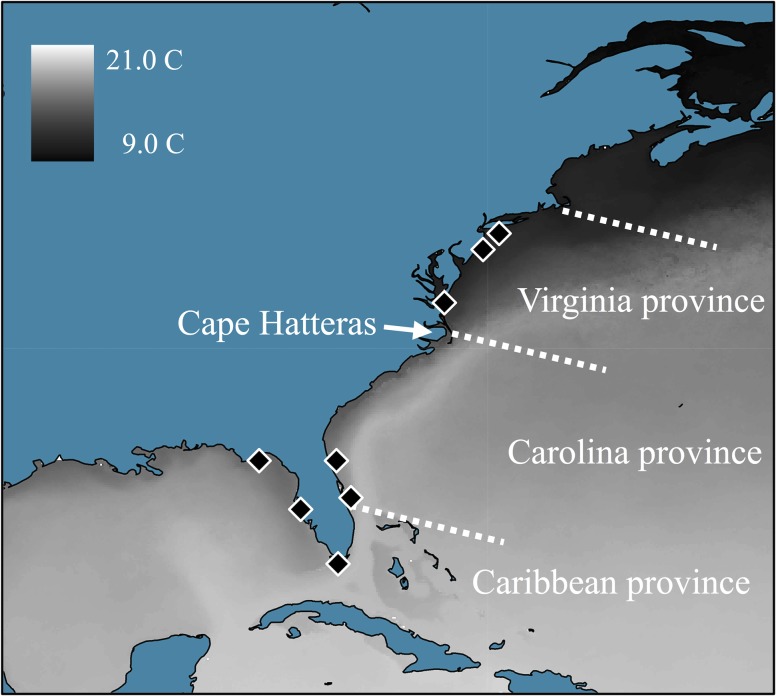
Map of zoogeographic provinces, collection sites, and temperature variance. Contrasting ocean minimum sea surface temperatures across zoogeographic provinces: generated in ARCGIS v.9.3 using the Bio-ORACLE long-term climatic dataset [67]. Collection sites from the northeastern Gulf of Mexico to New York State indicated by diamonds: Apalachicola, Tampa Bay-Charlotte Harbor, Florida Keys, Indian River Lagoon and Jacksonville, FL., Chesapeake Bay, New Jersey-New York.

In warmer seasons, the waters lining the concrete bulkheads, wooden piers, estuaries, and sandy beaches of the temperate Northeastern United State’s mid-Atlantic coast become home to numerous tropical fish species [1,2]. Over a century of research has cataloged the immigration of tropical vagrants or “strays” to these coastal mid-Atlantic waters. The majority of these individuals arrive due to passive planktonic dispersal in summer months, transported by ocean currents that circle north off the warm water mass of the Gulf Stream as it deflects northeast from U.S. towards Europe at roughly 35°N latitude [3,4]. This phenomenon positions Cape Hatteras as a delineation point between the zoogeographic Virginia and Carolina Provinces, each defined by distinct faunal endemism and unique macroclimatic conditions (Fig. 1) [5,6]. Following this observation, studies of species found in both provinces suggest that Cape Hatteras acts as a “barrier” where intraspecific gene flow is reduced between provinces or alternatively acts as a northern latitudinal limit during the winter for species without cold thermal tolerance [6,7]. In this latter case, more sedentary tropical species that passively drift into the temperate Virginia Province during warmer months locally perish after cold winter temperatures advance.

Though many fishes exhibit wide thermal tolerance, ascertaining the true range of marine species can be challenging due to factors that include patchy distributions, cyclical population sizes, and seasonal movement patterns [7,8]. One species often associated with tropical vagrants in the Virginia Province is the Lined seahorse, *Hippocampus erectus* [3]. Its status as a persistent independent gene pool (i.e., subpopulation) is uncertain primarily due to its nearshore absence during cold winter months and a scarcity of direct winter observations of individuals. *H. erectus* is commonly found in coastal zones in three zoogeographic provinces: Caribbean (tropical), Carolina (warm-temperate) and Virginia (temperate) (Fig. 1). Some researchers suggest that long-distance rafting carries migrants northward to temporarily inhabit the Virginia Province as temperatures warm [3], a prediction supported by substantial observational evidence of long-distance rafting migration throughout its range [9,10]. In contrast, other researchers suggest that localized active dispersal directed toward offshore migration for thermal refuge in continental shelf waters during late fall accounts for its winter absence [11]. This hypothesis of seasonal localized migration is partially supported by the observation of inshore colonization of *H. erectus* as temperatures warm in April to June, characteristic of most temperately adapted fishes [3], and earlier than the July to September arrival typical for the majority of tropical strays [1,12].

Ecologists and evolutionary biologists often focus on questions at different temporal scales, but both fields are increasingly making use of genetic data to test hypotheses about population history, estimate the movement of individuals between local populations, and characterize the spatial distribution of genetic variation for effective species management [8,13,14]. One example is the use of genetic data to examine source-sink dynamics [15,16]. True sink populations, even if annually persistent, require continual immigration from source populations and are expected to exhibit genetic homogeneity with source populations or heterogeneity reflecting multiple sources of immigrants, while over time independent breeding subpopulations through random (genetic drift) or deterministic (natural selection) processes will exhibit distinct genetic divergence [13]. A number of studies have examined the biogeography and genetic divergence of *Hippocampus* species [17]. Most of this research has focused on Indo-Pacific species with genetic variation ranging in spatial scales from among localized South African estuaries [18], to widespread species complexes associated with rafting driven colonization [19], and differing levels of intraspecific divergence attributed to both ecological traits and biogeographic divides [20,21].

Here we test whether the presence of *H. erectus* individuals north of Cape Hatteras are the result of an ephemeral deme that is seasonally replenished from demographically persistent southern populations (H1; Hypothesis 1), or in contrast, there are persistent and isolated populations on either side of Cape Hatteras (H2; Hypothesis 2). A previous study of the *H. erectus* complex utilized mitochondrial DNA and five more slowly evolving nuclear loci across many individuals (*n* = 115), yet rejected H2 in favor of H1, with little divergence and evidence of isolation across Cape Hatteras [22]. Now, with the decreasing cost of high-throughput sequencing, data can be sampled from across the autosomal genome to account for variations in mutation, coalescent history, and recombination, thereby facilitating a view of the complexity of a species evolutionary history with the potential to infer more recent divergence and/or populations differentiating in the presence of gene flow [23].

To date, genome wide single nucleotide polymorphism (SNP) datasets generated by restriction site associated DNA sequencing (i.e., RADseq) have been utilized to study several fish species. Examples utilizing RADseq datasets include the support of cryptic differentiation between populations of the Baltic Sea herring (*Clupea herangus*) [24], the detection of hybrid individuals between trout species [25], genetic divergence of various stickleback populations [26–28], and robust phylogenetic resolution between African cichlid species [29]. To test the aforementioned competing hypotheses H1 and H2, we generated a genomic RADseq dataset consisting of 11,708 SNPs across individuals of *H. erectus* from the eastern Gulf of Mexico to Long Island, NY (Fig. 1).

Although we base our inference from only 4–9 individuals per each of the three zoogeographic provinces (total individuals; *n* = 23), data from large numbers of unlinked loci allow highly resolved inference even with few individuals [30–32]. Moreover, given that outbred diploid genomes are comprised of recombining segments of DNA inherited from large pools of ancestors [33], genome-level datasets should capture the diversity of coalescent histories (across loci) that reflects population history, such that information comes more from the number of loci sampled through the genome than from numbers of individuals per sampling locality [34,35].

## Methods and Materials

### Sampling and bioinformatics

Samples of *H. erectus* ranged from the eastern Gulf of Mexico to New York State (*n* = 23). Samples were collected from 2009–2013 from the following locations: Apalachicola, FL, Tampa Bay, FL, Charlotte Harbor, FL, the Florida Keys, Jacksonville, FL, and Indian River Lagoon, FL, Chesapeake Bay, New Jersey, the Hudson River and Long Island, NY. The specimens collected in this study were carried out in accordance and approval of the Queens College Institutional Animal Care and Use Committee (IACUC) (Permit # 137), which approved all aspects of specimen use in this study. Domestic fishing of *Hippocampus* is neither under direct regulation within the United States nor under species protection and no specific permissions were required for these locations; however we collaborated with the following authorities for samples, and if standard collection permits were required they were issued for each collection location. The Florida specimens used in our study were collected under the authority of the Florida Fish and Wildlife (FFW) as part of the FFW: Southeast Area Monitoring and Assessment Program. Samples from the Chesapeake Bay were collected in collaboration with the Virginia Institute of Marine Science (VIMS), which is authorized to collect any fishes necessary for research under the Code of Virginia. Lastly, samples collected in New Jersey and New York were collected in collaboration with Rutgers University under the New Jersey Department of Environmental Protection and the New York State Department of Environmental Conservation (DEC) Special Licensing Unit, License No. 1638 with additional samples collected under DEC License No. 1405.

Sequenced samples were randomly chosen from a large number of individuals (*n* >100) over multiple collection years to ensure genomic similarity was not the result of non-independent relatedness. Total Genomic DNA was extracted using Puregene extraction (Qiagen) from tail muscle tissue and treated with RNAase A following standard protocols. Genomic DNA quality was checked on an agarose gel to ensure that the majority of DNA was >10,000bp and equalized to 30 ng/uL using Qubit Fluormetric Quantitation (Invitrogen). Library construction and restriction site associated DNA sequencing (RADseq) protocol followed [36,37]. Floragenex carried out library preparation and sequencing. Genomic DNA restriction digestion utilized the Sbfl enzyme and individual sequence adapters and barcode identifiers were ligated to genomic DNA prior to sequencing on the Illumina HiSeq platform. All sequences from cut sites resulted in single-end reads, which were demultiplexed and trimmed of adapters to 90bp fragment lengths.

Total reads per individual ranged from 1,264,862–4,736,299. The individual with the largest number of reads was processed to construct a de novo pseudo reference genome, and reads for each individual were aligned using BOWTIE [38]. SAMTOOLS algorithms [39] and custom Floragenex perl scripts were used to detect SNPs and call genotypes. SNP datasets were formatted in the variant call format (vcf) [40]. Initial genotyping required a minimum Phred quality score of 15, a minimum of 4× sequence coverage, with a minimum of 65% of individuals genotyped. Additional filtering was applied using R v.3 [41] to ensure a Phred score equal to a hard cutoff of q = 20 (base call accuracy lower than 99%). To reduce the inclusion of false SNP discovery due to paralogous sequences or low quality genotype calls, vcftools was utilized to remove any sites with a minimum depth of 8× sequence coverage and maximum depth calculated in R based on the mean depth + 1.5 standards deviation (= 295) across all sites. The final datasets resulted in a bi-allelic matrix of 11,708 genotypes (5777 90bp sequences) across individuals at all sites. For details on per individual raw reads, filtered, and analyzed reads see S1 Table.

### Population genomic analyses

**Figure 2:**
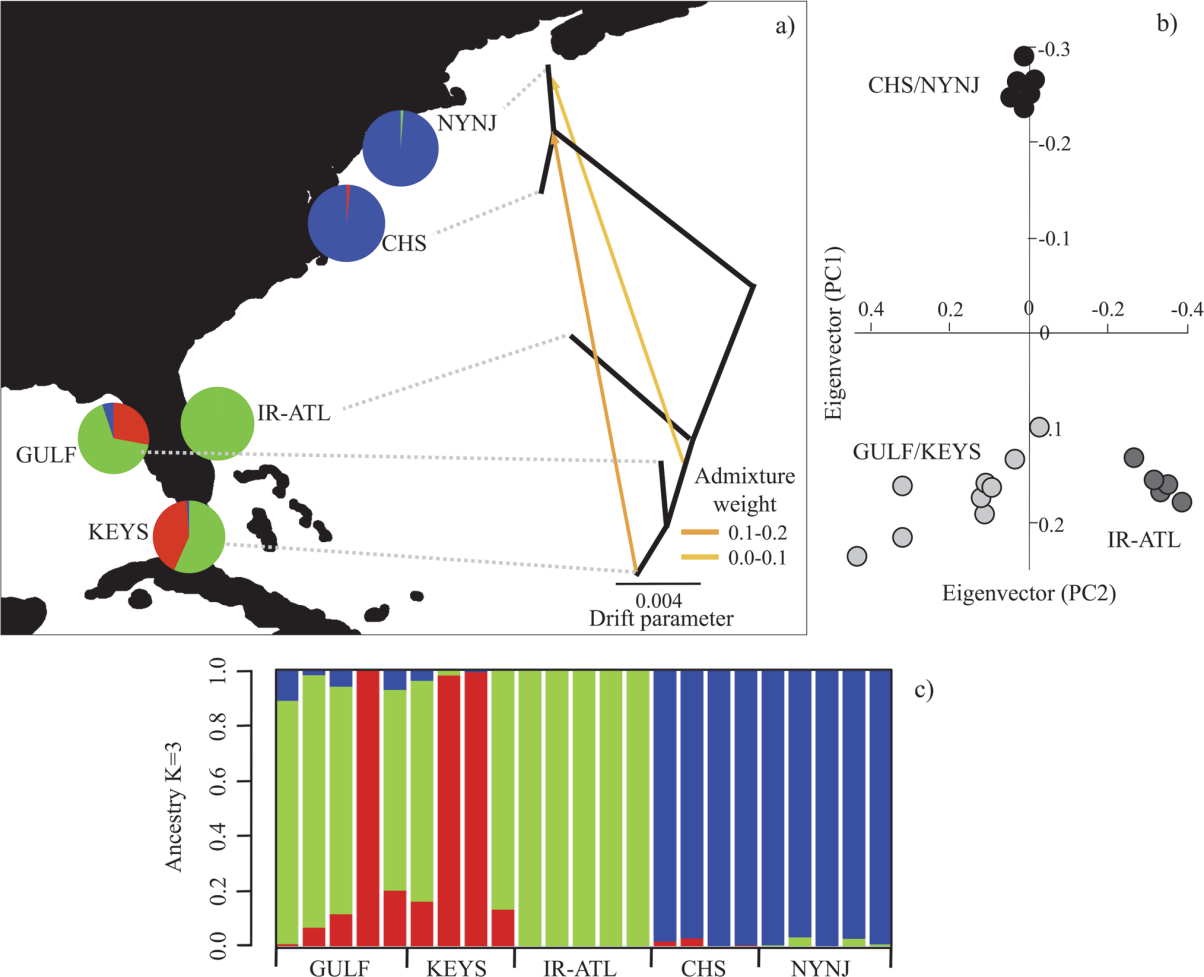
Genomic variation across individuals and subpopulations. (a) *Treemix* population tree with branch lengths scaled to the amount of genetic drift between regions and inferred proportion of genetic admixture (m = 2) between southern and northern regions represented by arrows. Dotted lines do not represent branch length. (b) Principle component analysis. Black circles = Chesapeake Bay-New York, dark grey circles = Florida Atlantic coast, and light grey circles = Gulf of Mexico-Florida Keys. Pie diagrams (a) represent ancestry coefficient proportions derived from the sNMF ancestry plot (c). Each line of the sNMF plot represents one individual.

A principal components analysis (PCA) was implemented to determine if sampled individuals reflect a history of differentiated populations by outputting individual coordinates along axes of genetic variation within a statistical framework [42] that correspond to the first two principle components in Fig. 2B. To further aide in assigning individuals to differentiated populations by inferring ancestry coefficients representing the proportions of each individual’s genome that originated from a specified number of ancestral gene pools (K) we used the program sNMF [43]. The program sNMF estimates individual ancestry and population clustering by utilizing a sparse non-negative matrix factorization algorithm (sNMF) to compute least-squares estimates of ancestry coefficients. This software is capable of efficiently analyzing large bi-allelic datasets without loss of accuracy when compared with more commonly utilized programs STRUCTURE [44] and ADMIXTURE [45] that use the same underlying model to infer ancestry coefficients. However, in contrast to the aforementioned programs, sNMF has significantly better computational efficiency and is robust to many of the demographic assumptions of Hardy-Weinberg and linkage equilibrium [43,46]. To verify the accuracy of this program Frichot et al. (2014) conducted an in-depth comparison with the software ADMIXTURE using simulated and empirical datasets and found concordant results across trials, while sNMF outperformed ADMIXTURE when population inbreeding (FIS) was high. For our dataset ancestry coefficients (K) were estimated using sNMF to determine subpopulation membership by running 10 replicates of K 2–6 using a cross-entropy criterion (CEC). To evaluate the predictive capability and error of the ancestry estimation algorithm, sNMF employs the CEC, which is comparable to the likelihood value implemented in the program ADMIXTURE. To select the best-supported ancestry coefficient, the lowest CEC value was represented by the K value (K = 3). The ancestry coefficient plot (Fig. 2C) was visualized using R v.3. For information on CEC values, as well as results obtained between sNMF and STRUCTURE on a subset of the total data (SNP = 2000) see S1 Methods.

The program *Treemix* [47] was utilized to infer the phylogenetic relationships between sampled locations while accounting for ancestral admixture among populations. Specifically, *Treemix* incorporates a model to allow for population divergence in the presence of post-divergence admixture/migration (m) given that incorporation of this parameter can improve the likelihood fit of a bifurcating phylogeny. More specifically, the m parameter represents the proportion of admixture from one population to another [48]. The resulting phylogeny is based on a composite maximum likelihood of the local optimum tree, determined using a similar approach to Felsenstein [49], with branch lengths proportional to the amount of genetic drift that has occurred per branch.

Population genetic statistics (Table 1; Fig. 2D-2F) were generated using vcftools and calculated across all SNPs per individual. The calculation of Fst utilized between subpopulations [50] specifically accounts for differences in sample size and a small number of sampled individuals, and recent studies have shown that bi-allelic SNPs (>1000) using this approach will result in precise Fst estimates [51].

**Table 1:** Population genetic summary statistics for each subpopulation.

**Subpopulations**	***n***	**Fst**		**SNPs**	**Mean He**	**Mean S**
**North-Atlantic**	**9**	**North-Atlantic**	**South-Atlantic**	**11,708**	**1,104**	**304**
**South-Atlantic**	**5**	**0.1012**	--------	**11,708**	**1,090**	**191**
**Gulf-Keys**	**9**	**0.083**	**0.0454**	**11,708**	**1,183**	**309**

Fst, number of individuals (*n*), total number of SNPs, observed mean heterozygote alleles per individual/per subpopulation (He) and mean number of singletons (S) per individual/per subpopulation.

To investigate the visual similarity between genetic and geographic distance from the PCA analysis (Fig. 2A and 2B), we conducted a test for isolation-by-distance (IBD) to see if this pattern meets the expectation of genetic similarity decaying with geographic distance [52] using the IBD program by Mantel’s test (10,000 randomizations) of linearized Fst (Fst/(1-Fst)) and shoreline distance (km) [53]. Pairwise Fst, calculated in vcftools, and distance of coastlines between sampling locations in kilometers was determined using Google Earth Tools. For this Mantel test, the centroid distance between sampling locations for the Gulf-Keys subpopulation was utilized and results indicated a non-significant correlation between geographic and genetic distance (p = 0.4925). See S1 Methods for additional information and regression plots.

## Results and Discussion

### Support for northern subpopulation divergence and isolation

Our results strongly support H2 over H1 with Virginia Province residents of *H. erectus* coming from a persistently breeding and isolated ancestral gene pool, rejecting the categorization of it being composed of seasonal migrants. The sNMF-based estimates of ancestry coefficients support three distinct subpopulations with limited admixture (K = 3) (Fig. 2C): 1) the eastern Gulf of Mexico-Florida Keys (Gulf-Keys), 2) the eastern Floridian Peninsula (South-Atlantic), and 3) Chesapeake Bay-New York (North-Atlantic). The K = 3 value reported in our study is considered robust as it exhibited the lowest CEC value across replicate runs of all K values (K = 2–6). This substructure also visually emerges from the first two principle components of the PCA from the total amount of observed genomic variation (Fig. 2B). Here, the individuals from north of Cape Hatteras form a tight cluster, while individuals sampled from the Gulf-Keys and South-Atlantic form a cline between the tightly clustered South-Atlantic individuals and an admixed set of Gulf-Keys individuals. Consistent with these results is the inferred population history that emerges from *Treemix*, which is concordant with long-term isolation of the North Atlantic sub-population with limited post-divergence admixture with southern subpopulations.

**Figure 3:**
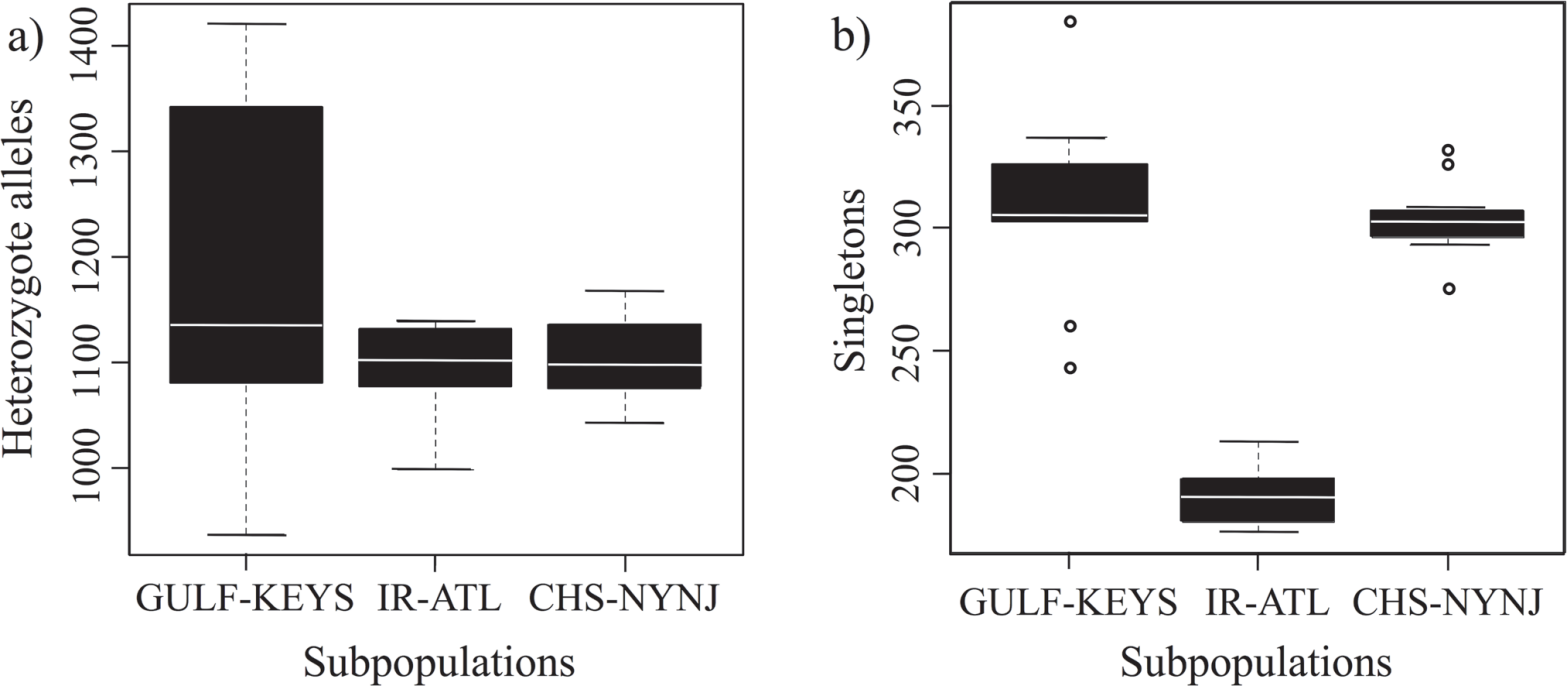
Distribution of heterozygote and singleton genotypes. Boxplots represent the range of observed heterozygote genotypes (a) and singleton genotypes per individual/per subpopulation (b).

The elevated heterozygosity found in Gulf-Keys individuals (Fig. 3A) could be the result of admixture from un-sampled western Gulf/Caribbean individuals, which is also indicated from the sNMF analysis (Fig. 2C). However, this elevated heterozygosity could also be the result of a larger effective population size [54]. In contrast, the northern subpopulation shows a reduction in heterozygosity with an elevated level of singletons (Fig. 3B). This pattern indicates a possible demographic expansion after the last glacial maximum that is consistent with the likely unsuitable habitat in the Virginia Province during the late Pleistocene. This history of shifting habitat driven by climate change is suggested by palaeo-climatological research indicating that temperate environments north of Cape Hatteras were displaced southward [6,55], as well as the formation of the Chesapeake Bay 7.4–8.2 kya due to post-last glacial maximum sea level rise [56].

### Causes of divergence and isolation of the northern subpopulation

Given the strong evidence we report for Virginia Province inhabitants of *H. erectus* representing a persistently isolated independent subpopulation from other regional ancestral gene pools, there are several conceivable non-mutually exclusive causes of this divergence. First, seagrass is a preferred breeding habitat of *H. erectus* and a long gap without coastal seagrasses exists along the Georgia and South Carolina coastlines (roughly 600km) [57]. This barrier of unsuitable breeding habitat between northern Florida and the Virginia Province therefore likely results in the fish’s rarity in this area, thereby increasing genetic isolation of the northern subpopulation [58,59]. The confamilial pipefish *Syngnathus floridae* also shares a similar pattern of genetic divergence across this region of unsuitability, though the area of absence extends from the southern end of the Florida Peninsula to near Cape Hatteras, with the northern population extending from North Carolina to Chesapeake Bay [60]. Secondly, long-distance migration of *H. erectus* is observed to occur via *Sargassum* rafting driven by ocean currents [10]. Under this mode of migration, the northeastern deflection in ocean currents near Cape Hatteras toward the Mid-Atlantic may limit the arrival of southern migrants to the Virginia province. Lastly, individuals that do arrive from southern provinces may have a lower physiological tolerance to temperate conditions, reducing the chance of winter survival and also increasing the amount of genetic isolation. Selection correlated to the shift in macroclimate at Cape Hatteras has been observed in marine fishes [61], and future analysis of northern adaptation in *H. erectus* may help decouple the potential drivers of temperate subpopulation genetic isolation. Although our observed patterns of genetic isolation could have emerged via a continuous isolation-by-distance regime without clear breaks driving the isolation, a Mantel test resulted in a non-significant relationship between genomic and geographic distance (*p* = 0.4925).

### Support for local seasonal migration

Our results also support local offshore migration to account for the coastal absence of *H. erectus* from Virginia Province during winter months. While extreme temperature changes influence latitudinal movement of many species [1], substantial seasonal movement to and from provinces for *H. erectus* is unlikely due to its relatively weak swimming ability [62]. To avoid nearshore cold water temperatures, localized inshore-offshore migration has been reported for the confamilial pipefish (*Syngnathus fuscus*), which has similar life history traits to *H. erectus* [63], and has also been suggested for some other species of *Hippocampus* [62]. As a qualitative comparison we examined abundance records from NOAA long-term offshore trawl surveys of *S. fuscus* (1972–2008; >90% 20 km off-coast; depth 10–20m) and found that they closely resemble that of *H. erectus*, further supporting intercontinental shelf overwintering (For additional details see S2 Methods). Regarding direct observation of this phenomena, a single record from divers in 1968 documented both species “hibernating” on the shelf substrates off Long Island, NY [64], where they resumed swimming several minutes after being brought to the surface. Many fish adapt to winter temperatures by decreasing energy demands and entering semi-torpidity [65], though no research has been conducted on the overwintering physiology of any Syngnathidae species. Nevertheless, localized overwintering in deeper waters may be an important component of *H. erectus*’ life history and may also account for their winter absence in estuaries of the warm-temperate eastern Floridian Peninsula (i.e., South-Atlantic).

## Conclusions

Overall, our results demonstrate the utility of supplementing life history information with population genomic data when a small number of unlinked genetic loci may be insufficient to discern the range of persistence in difficult-to-observe fishes. Currently, the IUCN (World Conservation Union) Red List categorizes *H. erectus* as “vulnerable” based on it being commonly collected as by-catch and sold by trawl fishermen to supply the aquarium trade [66]. Our results, throughout an extensive range of this species distribution, will help inform conservation, as well as captive breeding efforts, by strongly supporting northern Atlantic seahorses as a genetically distinct subpopulation. More broadly, because genomic data effectively samples a multitude of ancestors, even with a small number of sampled individuals, the approach taken in our study shows the promise of genomic data to infer population genetic structure in rare and/or difficult to obtain species.

## Supporting Information

S1 Table Raw reads, filtered, analyzed reads, and NCBI SRA accession numbers per individual. (PDF)

S1 Methods Additional details on sNMF CEC values, sNMF and STRUCTURE comparison, and isolation-by-distance methods. (PDF)

S2 Methods Comparison of NOAA Long-term bottom trawl survey of the Mid-Atlantic Bight (i.e., Virginia Province) between *Hippocampus erectus* and *Syngnathus fuscus*. (PDF)
