# Supplementary material for: Population Genomics Reveals Seahorses (Hippocampus erectus) of the Western Mid-Atlantic Coast to Be Residents Rather than Vagrants
Source: PLoS One. 2015 Jan 28;10(1):e0116219. doi: 10.1371/journal.pone.0116219 (PMC4309581; doi:10.1371/journal.pone.0116219)
Supplement: S1 Table — (PDF) [file pone.0116219.s001.pdf]

Table S1 Summary of genomic data collected for each individual and sampling location. Shown are the raw read counts per individual from the HiSeq Illumina run. The number of reads across individuals after initial processing and analyzed reads from homologous loci at all sites. Raw sequence reads are available at NCBI SRA Project: SRP048776.

Individual	SRA Accession Number	Location	Total 90bp reads	Reads post initial quality control (65% coverage, q=15)	Analyzed reads (100% coverage, q=20)
HEBGu1	SRX732943	Apalachicola, FL	3,157,866	1,110,944	814,411
HEGu2B	SRX732948	Apalachicola, FL	3,132,578	1,130,041	831,952
HETB1N	SRX732960	Tampa Bay, FL	1,338,653	534,152	398,006
HETB01	SRX732959	Tampa Bay, FL	2,578,857	973,553	712,973
HEBF1	SRX732942	Charlotte Harbor, FL	1,098,313	419,305	313,920
HEKY06	SRX732951	FL Keys	4,736,299	1,799,006	1,311,869
HEKY51	SRX732953	FL Keys	4,504,203	1,667,968	1,223,992
KYHE02	SRX732964	FL Keys	4,198,059	1,618,812	1,187,937
HEKY12	SRX732952	FL Keys	4,459,738	1,656,374	1,210,098
HEIR3	SRX732949	Indian River, FL	2,309,705	898,087	663,094
HEIR5	SRX732950	Indian River, FL	3,426,244	1,314,835	962,582
IR03HE	SRX732961	Indian River, FL	3,904,405	1,505,889	1,102,438
JXIR02HE	SRX732963	Jacksonville, FL	3,262,469	1,258,885	919,853
JXIR01HE	SRX732962	Jacksonville, FL	3,141,316	1,216,565	890,663
HECHS1	SRX732944	Chesapeake Bay	2,704,326	1,071,894	790,781
HECHS3	SRX732945	Chesapeake Bay	4,239,371	1,661,500	1,218,476
HECHS4	SRX732946	Chesapeake Bay	2,370,238	923,860	684,441
HECHS12	SRX732947	Chesapeake Bay	264,862	481,791	358,833
HENJ31	SRX732954	New Jersey	4,071,240	1,519,521	1,111,883
HENY05	SRX732955	New York	3,028,313	1,131,996	829,156
HENY09	SRX732956	New York	3,958,295	1,471,727	1,076,320
HENY20	SRX732957	New York	3,683,443	1,374,731	999,777
HENY29	SRX732958	New York	3,266,915	1,115,816	817,710
**Total Reads**			**72,835,708**	**27,857,252**	**20,431,165**
