# Supplementary material for: Population Genomics Reveals Seahorses (Hippocampus erectus) of the Western Mid-Atlantic Coast to Be Residents Rather than Vagrants
Source: PLoS One. 2015 Jan 28;10(1):e0116219. doi: 10.1371/journal.pone.0116219 (PMC4309581; doi:10.1371/journal.pone.0116219)
Supplement: S1 Methods — (PDF) [file pone.0116219.s002.pdf]

Methods S1: Additional details on the inference of ancestry coefficients, sNMF and STRUCTURE comparison test, and isolation-by-distance methods

Inference of ancestral gene pools: The program sNMF [1] was utilized to estimate individual ancestry and population clustering. Ancestry membership coefficients (K) were estimated to determine subpopulation membership by running 10 replicates of K 2-6 using a cross-entropy criterion (CEC). To select the ancestry coefficient with the highest likelihood, sNMF outputs a cross-entropy score, and the lowest value represents the best-supported K value (K=3) (Figure below).

Cross entropy score

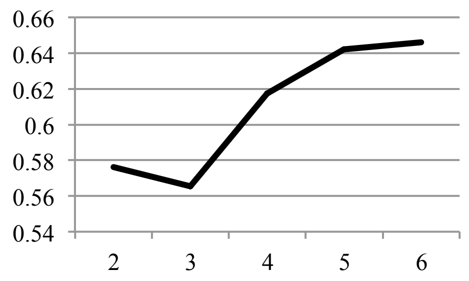

sNMF and STRUCTURE SNP subset comparison test: As a comparison test between STRUCTURE and sNMF we randomly selected 2000 SNPs from our full dataset. K-values ranging from 1 to 5 were analyzed in both programs with ten independent runs per K. In STRUCTURE each run consisted of 100,000 burn-in and 150,000 MCMC iterations, using the "Admixture Model" and "Correlated Allele Frequency Model" with default settings. This run length followed [2], and as they reported we found that results from longer preliminary runs were not different using more burn-in or MCMC iterations. STRUCTURE HARVESTER [3] and R were used to visualize the STRUCTURE results, with the most probable K chosen based on Delta-*K* [4]. The most probable K was selected based on cross-entropy criterion (CEC) values in sNMF. The CEC value can be interpreted as the inverse of Delta-*K*. Based on this subset of SNPs both programs supported the most probable K of 2. These plots clearly distinguish populations north and south of Cape Hatteras, though the differentiation of the cline visualized in our PCA and full data analysis separating eastern Gulf and southern Atlantic populations was not retained. Each line represents an individual from the following locations: GULF = Gulf of Mexico; KEYS = Florida Keys; IR-ATL = Jacksonville/Indian River Lagoon, FL; CHS = Chesapeake Bay; NYNJ = New York and New Jersey.

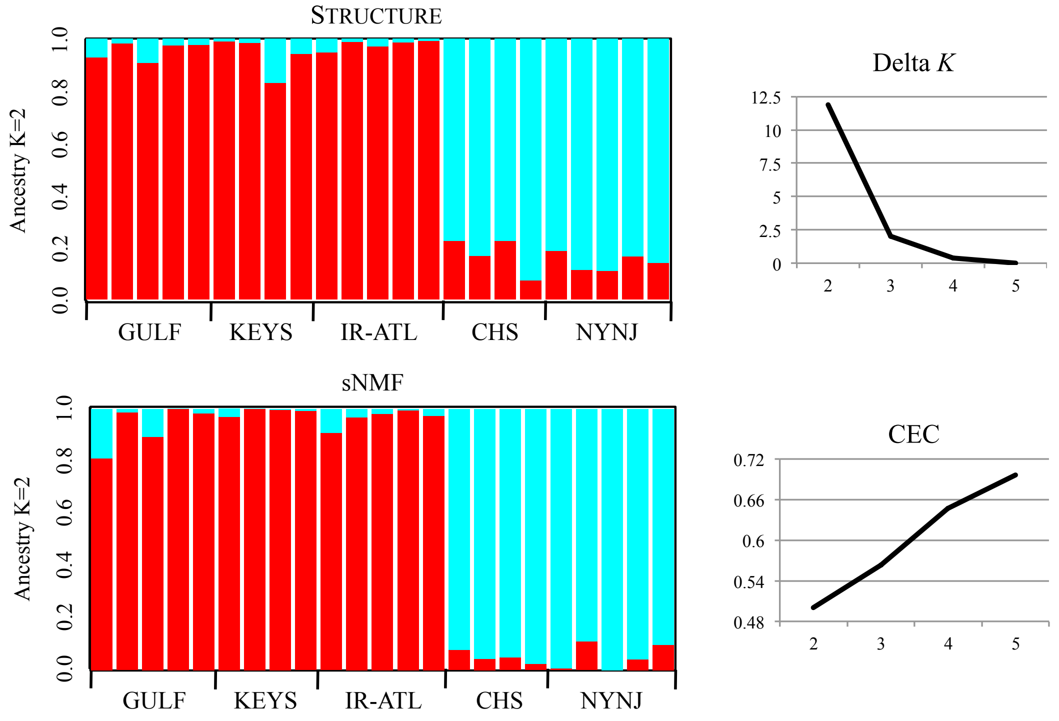

Isolation-by-distance: A test for isolation-by-distance was conducted using the IBD program [5] by Mantel's test (10,000 randomizations) of FST and shoreline distance (km). Pairwise linearized FST (FST /(1-FST) was calculated in vcftools and coastal distance of coastlines between sampling locations was determined using Google Earth Tools. For the between subpopulations test (a), as presented in the manuscript, the centroid distance between sampling locations for the Gulf-Keys subpopulation was utilized and results indicated a non-significant correlation between geographic and genetic distance (p = 0.4925). A second test for IBD between the five main sampling locations was also conducted (b) resulting in a non-significant value of p = 0.9026 (not reported in the manuscript).

a) b)

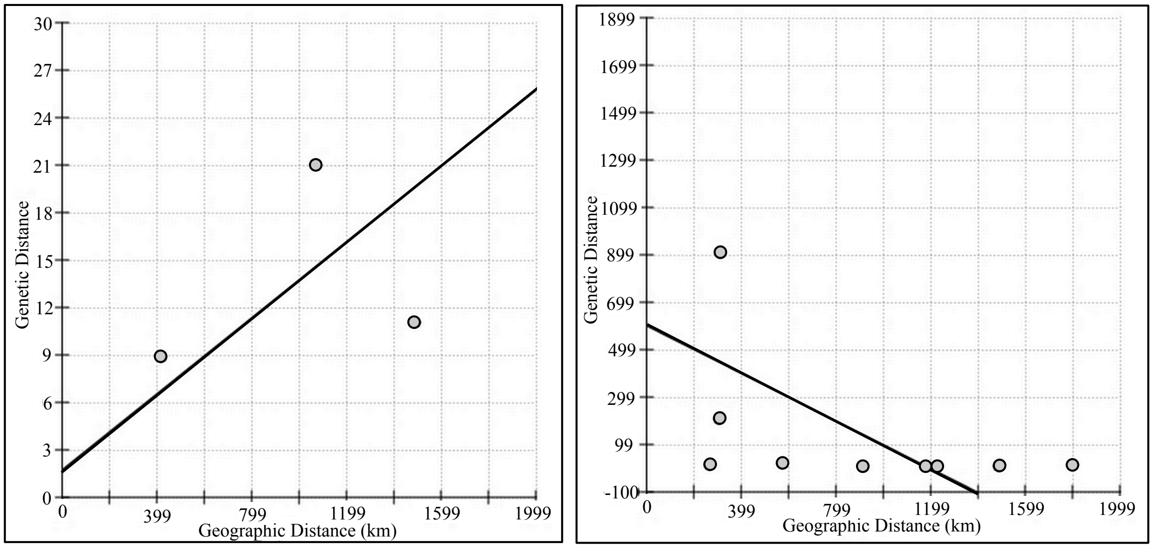

References

1. Frichot E, Mathieu F, Trouillon T, Bouchard G, François O (2014) Fast and efficient estimation of individual ancestry coefficients. Genetics 196: 973–983.

2. Massatti R, Knowles LL (2014) Microhabitat differences impact phylogeographic concordance of co-distributed species: genomic evidence in montane Sedges (*Carex*) from the Rocky Mountains. Evolution *Early View*.

3. Earl DA, vonHoldt BM (2011) STRUCTURE HARVESTER: a website and program for visualizing STRUCTURE output and implementing the Evanno method. Conserv Genet Resour 4: 359–361.

4. Evanno G, Regnaut S, Goudet J (2005) Detecting the number of clusters of individuals using the software STRUCTURE: a simulation study. Mol Ecol 14: 2611–2620.

5. Jensen J, AJ B, Kelley S (2005) Isolation by distance, web service. BMC Genet 6: http://ibdws.sdsu.edu/.
