# Supplementary material for: Population Genomics Reveals Seahorses (Hippocampus erectus) of the Western Mid-Atlantic Coast to Be Residents Rather than Vagrants
Source: PLoS One. 2015 Jan 28;10(1):e0116219. doi: 10.1371/journal.pone.0116219 (PMC4309581; doi:10.1371/journal.pone.0116219)
Supplement: S2 Methods — (PDF) [file pone.0116219.s003.pdf]

Methods S2: NOAA Long-term bottom trawl survey of the Mid-Atlantic Bight (i.e., Virginia province)

Aggregated monthly catch totals from the Virginia province conducted from 1972-2008 for *Hippocampus erectus* and *Syngnathus fuscus.* Peaks in monthly catch totals of *H. erectus* correspond to inshore-offshore local intercontinental shelf migration reported for *S. fuscus*. For additional details on specific migration rates of *S. fuscus* across latitudes see [1]. >90% of samples were collected off the coast of Chesapeake Bay to Cape Cod, MA., at a depth 10-20m. Dependent on latitude the majority of both species are found in nearshore zones and estuaries between the months of April-October, with higher latitudes resulting in earlier off-shore catch abundance.

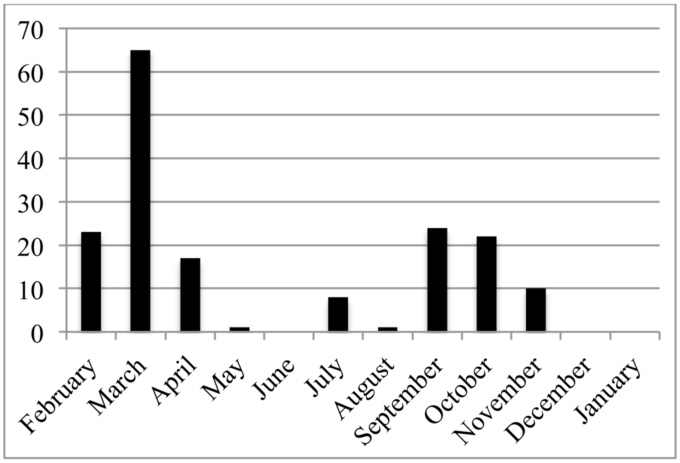

H. erectus monthly non-coastal catch totals from 1972-2008

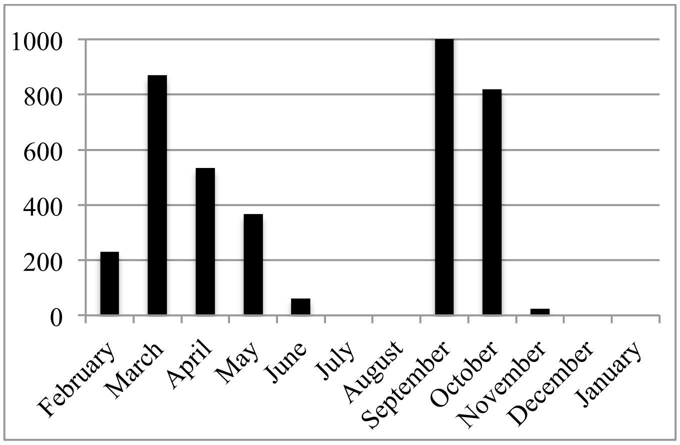

S. fuscus monthly non-coastal catch totals from 1972-2008.

References

1. Lazzari MA, Able KW (1990) Northern pipefish, Syngnathus fuscus, occurrences over the Mid-Atlantic Bight continental shelf: evidence of seasonal migration. Environ Biol Fishes 27: 177–185.
